# Comment on Nascimento et al. Citations Network Analysis of Vision and Sport. *Int. J. Environ. Res. Public Health* 2020, *17*, 7574

**DOI:** 10.3390/ijerph18126488

**Published:** 2021-06-16

**Authors:** Daniel M. Laby, Lawrence G. Appelbaum

**Affiliations:** 1 ChampionsEdge, LLC, New York, NY 10022, USA; 2 Department of Psychiatry and Behavioral Sciences, Duke University School of Medicine, Durham, NC 27710, USA; greg@duke.edu

In October 2020, the paper “Citations Network Analysis of Vision and Sport [1]” was published in the *International Journal of Environmental Research and Public Health*. Using the Web of Science database, the authors used several search terms (sport, vision, and eye) to identify sports vision publications, the most frequently cited publication, as well as the journals that published the most articles in sports vision, among other measures. Although publications by authors in a growing field are critical to the growth of that field, the accuracy and completeness of the publications are perhaps more critical.

As frequent contributors to the field of sports vision, and with over half a century of combined experience, we read, with great interest, the above publication, and looked forward to gaining a greater understanding of the scope and breath of publications. Unfortunately, after critically reviewing the manuscript, we noted several potential fatal flaws in the methodology and resulting interpretation of results by the authors.

Perhaps, and possibly most importantly, the authors failed to include many publications in the field of sports vision. This may have been caused by the very narrow search terms used in the initial Web of Science search, as well as restricting the search to a single database. For example, a PubMed search for the terms, “sport” and “eye” reveals ~3300 citations. A larger number is noted in a search of the Google Scholar database. The use of only three keywords also removed from the analysis the many publications that do not include those terms. For example, a search of the PubMed database with the terms “baseball” and “eye” revealed 142 publications, a search of “soccer” and “eye” revealed 148 citations and a search of “rugby” and “eye” revealed 182 publications, as examples.

When conducting a survey of the literature, with the intention of noting the frequency of publications, authorship as well as journal frequency, it is imperative that the pool of analyzed publications be as complete as possible and be created by multiple search criteria, as well as multiple search engines. It appears that the authors did not conduct a sufficiently comprehensive search of the sports vision literature; thus, severely under-representing the field, and as a result skewing their results and interpretation.

The restrictive analysis created several issues with regard to study results. For example, Table 4 in the manuscript, listing the “top 10 authors with the largest number of publications” notes Mann, D.L. as the author “with the largest number of publications in sports vision” with a total of 10, and at the other end of the table, they note Kredel, R. as having three publications. A count of all the sports vision papers authored by Mann, D.L., listed in PubMed, totals about four-times the number listed in the table. Likewise, there are many authors in the sports vision field who have published many more publications than those listed in the “top 10” table in this publication (e.g., Gray, R., Abernethy, B., Laby, D.M., Kirschen, D., Williams, M., Appelbaum, L.G., etc.).

The authors note that the most cited publication in the field of sports vision was by Williams et al. in 2002 regarding Quiet Eye duration. The authors note that this article was cited 55 times. We found it interesting that the creator of the “Quiet Eye” concept, Prof Joan Vickers, was not even included in the list of top 10 authors, despite her articles in the field having been cited hundreds of times each. For example, her 1996 article, titled “Visual control when aiming at a far target [2]”, which escaped the keyword search by the authors despite describing the gaze characteristics of basketball athletes, has been cited more than 600 times. Additionally, many other of her foundational publications, that have been cited hundreds of times, were not included in the author’s publication, most likely because of the very narrow and incomplete search of the literature.

Lastly, the authors failed to include, or make note of, recent literature reviews in the field of sports vision, which would have been helpful in their data acquisition and resulting analysis. For example, in a 2016 publication, Appelbaum and Erickson [3] reviewed different sports vision training interventions. In that publication, the authors cite almost 175 publications relevant to their review. Clearly, the overwhelming majority of these publications, although certainly overlapping with other fields, should have been considered by the authors as publications in the field of sports vision. Interestingly, the Web of Science lists 59 citations for this publication, more than double the number noted by the authors for the publication by Williams [4]. Moreover, the first two words in the title of this article are “Sports Vision” opening questions as to how this paper escaped the keyword search performed in this citation network analysis.

As the authors correctly note, sports vision is a relatively new specialty. A specialty that is comprised of a varied and cross-disciplinary population of ophthalmologists, optometrists, as well as vision scientists and other researchers and practitioners. The growing field depends on the scientific study and reporting of data, which is completed in adherence to scientific norms, is well executed, and properly reviewed prior to publication to be of benefit to the field. We commend the authors on their intention to contribute to the field but note several serious flaws that indicate this is a poor representation of the desired literature. We would encourage the authors to conduct a more comprehensive review but in the interest of completeness, we crafted this letter so that readers are aware of the shortcomings in this published article.
